# Lateral ankle ligament reconstruction using free split peroneal brevis tendon and fibula bone pegs: A case report

**DOI:** 10.1016/j.ijscr.2025.111955

**Published:** 2025-09-18

**Authors:** Ryogo Furuhata, Yuki Yamai, Yoshihiko Kamikawa, Atsushi Tanji

**Affiliations:** Department of Orthopaedic Surgery, Ashikaga Red Cross Hospital, Ashikaga-shi, Tochigi, Japan

**Keywords:** Ankle, Bone peg, Fibula, Lateral ligament reconstruction, Peroneal tendon, Case report

## Abstract

**Introduction:**

Ankle sprain is a frequent soft-tissue injury; however, chronic pain sometimes persists, requiring surgery. Tenodesis repair using free tendon graft provides satisfactory outcomes in cases where no viable ligament structure is available. However, rupture of the graft tendon can occur postoperatively, causing recurrent ankle instability. We report a case of lateral ligament reconstruction using fibula bone pegs to enhance the graft tendon fixation strength for a chronic calcaneofibular ligament injury.

**Presentation of case:**

A 44-year-old male presented with right ankle pain that had persisted since a fall one year earlier. Magnetic resonance imaging revealed a rupture of the right calcaneofibular ligament. Conservative treatment failed to improve pain, necessitating surgery. We made a curvilinear incision over the lateral ankle and harvested the anterior half of the peroneus brevis tendon. A bone tunnels of the fibula and the calcaneus was created, and the graft tendon was passed through it. Two bone pegs were harvested from the distal fibula, and inserted into the bone tunnel to fix the graft tendon. Postoperatively, ankle pain improved without any complications.

**Discussion:**

Lateral ligament reconstruction using fibula bone pegs provided satisfactory short-term outcomes. The use of fibula pegs, rather than special synthetic implants, allows for secure fixation of the tendon using the patient's own bone and eliminates the risk of foreign body reactions.

**Conclusion:**

This report presents a new fixation procedure on the lateral ankle ligament reconstruction using free graft tendon, which can improve physiological fixation strength of the graft tendon.

## Highlights

• This case report presents a novel fixation procedure on the lateral ankle ligament reconstruction
• A free graft tendon was fixed with bone pegs harvested from the fibula
• The use of fibula bone pegs can improve physiological fixation strength of the graft tendon

## 1 Introduction

Ankle sprain is a common orthopaedic injury; however, even when appropriately treated nonoperatively, chronic lateral ankle pain and instability may persist in 10–20 % of cases [1]. When lateral ankle pain persists for more than 6 months, surgery is generally indicated [2]. To date, various surgical techniques have been reported for repairing the lateral ankle ligament injury. In cases where anatomical reconstruction using remnants is difficult due to the extended period since injury, tenodesis stabilization can be indicated [[3], [4], [5]]. Tenodesis repairs using a free tendon graft have yielded satisfactory mid- and long-term postoperative outcomes, especially in cases where no viable ligament structure is available [[3], [4], [5]]. However, these tenodesis procedures may result in complications such as postoperative rupture of the graft tendon or disruption of the tenodesis [[6], [7], [8], [9], [10], [11]]. These complications can cause recurrent lateral ankle instability [12,13]; therefore, measures to prevent these complications have been reported that enhance graft tendon fixation strength by using interference screws or suture anchors [[13], [14], [15], [16], [17]].

On the other hand, in the surgery for ulnar collateral ligament injuries of the elbow joint, reconstruction using bone pegs of olecranon to fix the graft tendons has reported satisfactory postoperative outcome [18]. However, there are no reports of bone peg fixation applying to the surgery for chronic ankle instability.

Here, we report a case of lateral ligament reconstruction using a free split peroneal brevis tendon and fibula bone pegs for a chronic calcaneofibular ligament injury. This work has been reported in line with the SCARE criteria [19].

## 2 Presentation of case

**Fig. 1:**
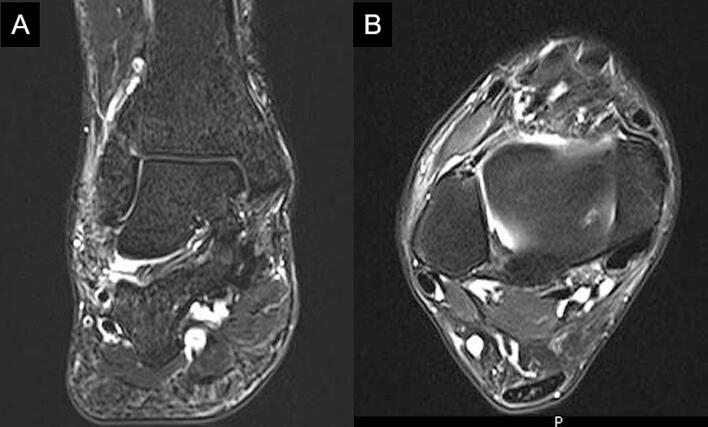
Magnetic resonance imaging of the right ankle shows a rupture of the right calcaneofibular ligament in the fat-suppressed T2-weighted coronal image (A) and an injury of the right anterior talofibular ligament in the fat-suppressed T2-weighted axial image (B).

A 44-year-old male presented to our hospital with right ankle pain that had persisted since a fall with an inversion mechanism one year earlier. He underwent conservative treatment using a brace for a month post-injury; however, right ankle pain persisted. He had a history of panic disorder. Tenderness was noted in the right calcaneofibular ligament and anterior talofibular ligament, and his pain worsened with plantarflexion and dorsiflexion of the ankle joint. The American Orthopaedic Foot and Ankle Society (AOFAS) score was 53. Radiography showed no abnormal findings. Magnetic resonance imaging revealed a rupture of the right calcaneofibular ligament and an injury of the right anterior talofibular ligament (Fig. 1A and B). Conservative treatment failed to improve pain, leading to the decision for surgery.

**Fig. 2:**
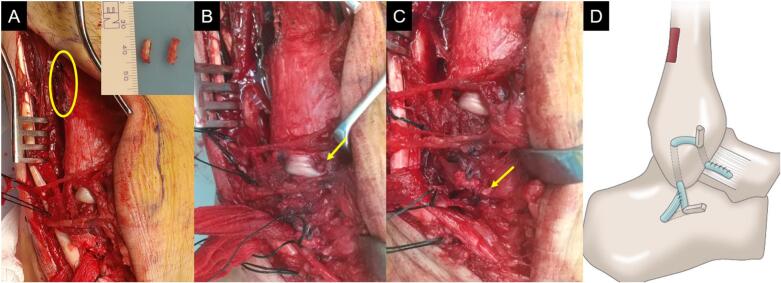
Intraoperative findings of the right lateral ankle ligament reconstruction. After passing the graft tendon through the bone tunnels, we harvested two bone pegs from the distal fibula (yellow circle) (A). We inserted a bone peg (yellow arrow) into the bone tunnel created at the insertion site of the anterior talofibular ligament of the fibula to fix the graft tendon (B). Additionally, we inserted the other bone peg (yellow arrow) into the bone tunnel created in the calcaneus with tension applied to the graft tendon (C). The illustration of lateral ligament reconstruction using a free split peroneal brevis tendon and fibula bone pegs for a chronic calcaneofibular ligament injury.

**Fig. 3:**
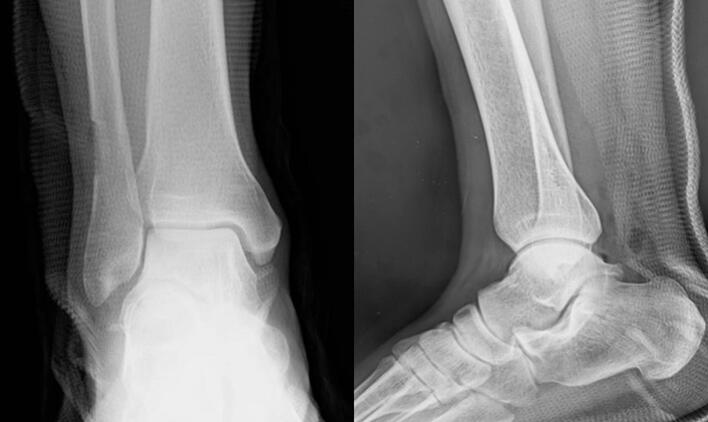
Radiograph taken immediately after surgery.

We performed the reconstruction using the free split peroneus brevis tendon according to the procedure described by Hashimoto et al. [5]. A curvilinear skin incision was made from the distal end of the fibula to the base of the fifth metatarsal bone. The rupture of the calcaneofibular ligament was identified and the continuity of the anterior talofibular ligament was preserved. After the peroneus brevis tendon was exposed, we harvested the anterior half of the tendon from the distal end of the base of the fifth metatarsal bone to the musculotendinous junction. A 10 cm segment of the split peroneus brevis tendon was used as the graft tendon. Subsequently, two bone tunnels were created at the insertion site of the anterior talofibular ligament and the calcaneofibular ligament of fibula with a diameter of 3.5 mm drill. Additionally, a 1 cm bone tunnel was created in the calcaneus at the insertion site of the calcaneofibular ligament. After passing the graft tendon through the bone tunnels, two bone pegs were harvested from the distal fibula, 1 cm in length (Fig. 2A). First, the ends of the graft tendon were sutured to the anterior talofibular ligament using 2–0 non-absorbable sutures and inserted a bone peg into the bone tunnel created at the insertion site of the anterior talofibular ligament on the fibula to fix the graft tendon (Fig. 2B). Next, with the ankle in a neutral position, we inserted the other bone peg into the bone tunnel created in the calcaneus with tension applied to the graft tendon (Fig. 2C). The free ends of the graft tendon were folded back at the bone hole in the calcaneus and sutured side-to-side using 2-0 nonabsorbable sutures (Fig. 2D). Postoperative radiographs are shown in Fig. 3.

The affected limb was kept non-weight-bearing for 4 weeks postoperatively, with the ankle fixed in a short leg cast. Full weight-bearing was permitted 4 weeks postoperatively. Heavy labor was permitted at 3 months postoperatively. The ankle range of motion (right/left) was 45/45° for dorsiflexion, 45/45° for plantarflexion, and 35/40° for introversion at 10 months postoperatively. The AOFAS score was 85 and the visual analog scale score was 1 at 10 months postoperatively.

## 3 Discussion

In this case, lateral ligament reconstruction using a free split peroneal brevis tendon and fibula bone pegs provided satisfactory short-term functional outcomes without complications for a chronic calcaneofibular ligament injury.

In conventional reconstruction procedure using peroneal tendon, the fixation strength of the graft tendon depends on the suture or tendon strength. Recent studies have developed the procedures using interference screws or suture anchors to achieve sufficient strength at the tendon graft-bone tunnel junction [[13], [14], [15], [16], [17]]. The modified Brostrom–Evans procedure secured with interference screws has produced good functional outcomes and a low complication rate [14,16]. The functional outcomes of our case were comparable to those reported in previous studies [14,16]. Tenodesis stabilization carries the risk of postoperative limitation of range of ankle motion; however, in this case, the postoperative range of motion was nearly identical to that of the unaffected side.

This surgical procedure offers two advantages over reconstruction techniques using interference screws or suture anchors. First, the use of a fibular peg, rather than special synthetic implants, allows for secure fixation of the tendon using the patient's own bone. This prevents a decrease in bone strength in the calcaneus or fibula, in which the graft tendon was harvested. A previous study on the biomechanical strength of bone pegs in phalangeal fracture models showed that bone peg fixation demonstrated significantly better rigidity than did fixation using bioabsorbable rods alone in bending, torsion, and failure tests [20], supporting our hypothesis. Second, bone pegs are autogenous, eliminating the risk of foreign body reactions that are associated with synthetic polymers [[12], [13], [14], [15], [16], [17], [18], [19], [20], [21], [22], [23]].

In addition, in a biomechanical study on the bone fusion effects of bone and metal pegs, the push-out force at 14 days after inserting these pegs into the rabbits' femur was higher in bone peg fixation than in metal peg fixation, suggesting that early bone-to-bone fusion with bone pegs is superior to bone-metal contact with metal implant [24]. These biomechanical results suggest that fibula bone pegs enable early physiological bone-tendon fusion between the bone tunnel and the graft tendon. Reconstruction using the palmaris longus tendon and olecranon bone peg for ulnar collateral ligament injury has yielded excellent outcomes with a high return-to-sports rate in baseball players [18]; however, there is no report which applied this technique using bone pegs to fix the graft tendon to chronic lateral ankle instability. Further biomechanical studies and comparative studies are required.

However, the risk of iatrogenic fracture during bone peg removal is a drawback of this technique. To prevent this complication, we first created bone holes around the harvesting site using a Kirschner wire and subsequently harvested the fibula bone pegs using a chisel. Additionally, we used fluoroscopy to confirm that the bone pegs were harvested proximal to the inferior tibiofibular ligament, which plays a role in transmitting weight bearing [25].

This case report has some limitations. First, long-term outcomes were not evaluated in this case; however, tenodesis repairs using a free tendon graft may cause osteoarthritis and loss of stability over time [26,27]. Second, this is a single-case report, limiting the generalizability of the findings. A comparative study with a larger number of cases and long-term outcomes is warranted to evaluate the usefulness of this procedure.

## 4 Conclusion

This case report presents a new fixation procedure on the lateral ankle ligament reconstruction using free graft tendon. The use of fibular bone pegs can contribute to physiological fixation strength of the graft tendon for chronic lateral ankle instability.

## Abbreviations

AOFAS: American Orthopaedic Foot and Ankle Society

## CRediT authorship contribution statement

RF and YY were responsible for conceptualization, data curation, formal analysis of the clinical data, and wrote the manuscript. YK and AT was responsible for formal analysis, interpreted the clinical data, provided clinical advice, and critically revised the manuscript for important content. All of the authors have read and approved the final manuscript.

## Consent

Written informed consent was obtained from the patient for the publication of this case report and any accompanying images. A copy of the written consent form is available for review.

## Ethical approval

Approval from the local ethics committee was not required for this anonymized case report in accordance with legislation enforced by the Institutional Review Committee of Ashikaga Red Cross Hospital.

## Guarantor

Ryogo Furuhata.

## Research registration number

1. Name of the registry: Not applicable
2. Unique identifying number or registration ID: Not applicable
3. Hyperlink to your specific registration: Not applicable.

## Funding

This research did not receive any specific grant from funding agencies in the public, commercial, or not-for-profit sectors.

## Declaration of competing interest

None of the authors have any conflicts of interest to declare.

## Data Availability

Data supporting the findings of this study are available from the corresponding author upon request.
